# Implementing a Novel Quality Improvement-Based Approach to Data Quality Monitoring and Enhancement in a Multipurpose Clinical Registry

**DOI:** 10.5334/egems.262

**Published:** 2019-09-30

**Authors:** Jesse Pratt, Daniel Jeffers, Eileen C. King, Michael D. Kappelman, Jennifer Collins, Peter Margolis, Howard Baron, Julie A. Bass, Mikelle D. Bassett, Genie L. Beasley, Keith J. Benkov, Jeffrey A. Bornstein, José M. Cabrera, Wallace Crandall, Liz D. Dancel, Monica P. Garin-Laflam, John E. Grunow, Barry Z. Hirsch, Edward Hoffenberg, Esther Israel, Traci W. Jester, Fevronia Kiparissi, Arathi Lakhole, Sameer P. Lapsia, Phillip Minar, Fernando A. Navarro, Haley Neef, KT Park, Dinesh S. Pashankar, Ashish S. Patel, Victor M. Pineiro, Charles M. Samson, Kelly C. Sandberg, Steven J. Steiner, Jennifer A. Strople, Boris Sudel, Jillian S. Sullivan, David L. Suskind, Vikas Uppal, Prateek D. Wali

**Affiliations:** 1 Pharmaceutical Product Development, US; 2 Total Quality Logistics, US; 3 Cincinnati Children’s Hospital Medical Center, University of Cincinnati, US; 4 University of North Carolina at Chapel Hill, US; 5 Cincinnati Children’s Hospital Medical Center, US; 6 Pediatric Gastroenterology & Nutrition Associates, US; 7 Children’s Mercy, US; 8 OHSU Doernbecher Children’s Hospital, US; 9 UF Health Pediatric Gastroenterology, Hepatology and Nutrition, US; 10 Kravis Children’s Hospital at Mount Sinai, US; 11 Arnold Palmer Hospital for Children, US; 12 Children’s Hospital of Wisconsin, US; 13 Eli Lilly and Company, US; 14 Greenville Health System, Children’s Hospital, US; 15 Carilion Clinic Children’s Hospital, US; 16 Oklahoma University Medical Center, US; 17 Baystate Medical Center, US; 18 Children’s Hospital Colorado, US; 19 MassGeneral Hospital for Children, US; 20 Children’s of Alabama, US; 21 Great Ormond Street Hospital, GB; 22 UCSF Benioff Children’s Hospital Oakland, US; 23 Children’s Hospital of the King’s Daughters, US; 24 Children’s Memorial Hermann Hospital – UT Houston, US; 25 University of Michigan – C.S. Mott Children’s Hospital, US; 26 Genentech, US; 27 Yale-New Haven Children’s Hospital, US; 28 UT Southwestern/Children’s Health, US; 29 Levine Children’s Hospital, US; 30 St. Louis Children’s Hospital – Washington University, US; 31 Dayton Children’s Hospital, US; 32 Riley Hospital for Children, US; 33 Ann and Robert H. Lurie Children’s Hospital of Chicago, US; 34 University of Minnesota, US; 35 The University of Vermont Children’s Hospital, US; 36 Seattle Children’s Hospital, US; 37 Nemours Children’s Health System – Wilmington, US; 38 Upstate Golisano Children’s Hospital, US

**Keywords:** Quality Improvement, Data Quality, Registry

## Abstract

**Objective:**

To implement a quality improvement based system to measure and improve data quality in an observational clinical registry to support a Learning Healthcare System.

**Data Source:**

ImproveCareNow Network registry, which as of September 2019 contained data from 314,250 visits of 43,305 pediatric Inflammatory Bowel Disease (IBD) patients at 109 participating care centers.

**Study Design:**

The impact of data quality improvement support to care centers was evaluated using statistical process control methodology. Data quality measures were defined, performance feedback of those measures using statistical process control charts was implemented, and reports that identified data items not following data quality checks were developed to enable centers to monitor and improve the quality of their data.

**Principal Findings:**

There was a pattern of improvement across measures of data quality. The proportion of visits with complete critical data increased from 72 percent to 82 percent. The percent of registered patients improved from 59 percent to 83 percent. Of three additional measures of data consistency and timeliness, one improved performance from 42 percent to 63 percent. Performance declined on one measure due to changes in network documentation practices and maturation. There was variation among care centers in data quality.

**Conclusions:**

A quality improvement based approach to data quality monitoring and improvement is feasible and effective.

## Introduction

There is growing interest in the potential for clinical registries that can simultaneously support clinical care, quality improvement (QI), and research. This multi-purpose model is consistent with the Institute of Medicine’s (IOM’s) vision of a Learning Health System which “draws research closer to clinical practice by building knowledge development and application into each stage of the health care delivery process” [1]. Gliklich and Dreyer [2] define a registry as “an organized system that uses observational study methods to collect uniform data (clinical and other) to evaluate specified outcomes for a population defined by a particular disease, condition, or exposure, and that serves one or more predetermined scientific, clinical, or policy purposes.” Most pediatric chronic illnesses meet the NIH definition for rare disease [3] and, as such, multi-center registries are especially important to study and improve care for children with chronic diseases. Some multi-center networks are beginning to adopt principles of open science, or network-based production [4], to foster collaborative improvement, research, data sharing, and innovation. In this setting, the registry functions not only to provide access to condition-specific information in a uniform way to support clinical care but also to support QI and research to improve patient outcomes.

The challenges and opportunities in managing data from multi-purpose clinical registries that are used for care, QI, and research are distinct from those that arise in the management of data collected specifically for study purposes, particularly clinical trials. This is largely due to the differences in the purpose of and resources available for data collection. In clinical trials, data collection involves a limited and pre-specified number of participants (based on a sample size determination). Data collection occurs at pre-specified time intervals (i.e. study visits) for a defined period of time. In addition, the trial data collection system is closed at the end of the study. In contrast, registries are designed to support real time care, quality improvement, and knowledge development. They involve data collection as part of routine care and must embed the process of data collection into the clinical workflow. The data reflect actual practice and patient care. Challenges in this setting may include data collection at every patient visit over an extended period of time, unstandardized visit schedules, and large numbers of data elements needed to support chronic care activities such as population management [5] and pre-visit planning [6] for an entire patient population. In addition, care centers participating in multi-purpose registries participate voluntarily. Many members of the clinical care team are involved, and resources for data capture and cleaning, such as clinical auditing and source document verification, are substantially less compared with clinical trials. The same staff responsible for transcribing data from the medical record and entering into the electronic case report forms may also be responsible for completing source document verification, in addition to other administrative and/or clinical responsibilities. Such systems cannot support the data cleaning efforts typical of clinical trials that involve large numbers of queries sent to care centers for response. A key challenge to using data from registries for research is that the quality may not match that of data collected using other, more rigorous and expensive, study support [7]. To date, studies of data quality in registries have focused on retrospective assessments of the “fit for use” model which indicates that the data quality is appropriate for the intended use [8,9].

Multi-center registries have used quality improvement methodology to improve patient care and outcomes. These same methods may be extended to interventions that enable teams to improve data quality. The impact of a data quality improvement project based on good clinical data management practices [10] was evaluated within a multi-center registry for clinical care, QI, and research.

## Methods

### Setting and Centers

The ImproveCareNow (ICN) Network (www.improvecarenow.org) is a multi-center international research and quality improvement network whose purpose is to transform the health, care, and costs for all children and adolescents with inflammatory bowel disease (IBD), specifically Crohn’s disease and ulcerative colitis. The network seeks to enable patients, families, clinicians and researchers to work together to accelerate innovation, discovery and the application of new knowledge. All 74 participating care centers entering data in the ICN registry from June 2010 through June 2016 were included in this study, representing data from 162,626 visits from 24,309 patients.

The design of the Network has been described in detail previously [11,12]. Briefly, ICN care centers include a mix of large and small academic medical centers and private practices over diverse geographic regions (urban/rural) and include approximately 60 percent of all pediatric gastroenterologists in the United States. Providers at each center receive instruction and ongoing coaching in QI methods, use of tools, and performance reports. Monthly webinars and semi-annual Community Conferences are held to provide ongoing training. Participating clinicians have developed care guidelines, tools, and processes to reduce variation in care. Centers within the network collect standardized data elements at the time patients are enrolled into the registry, at all follow-up visits, in the event of a hospitalization, and when the patient discontinues participation in the registry. These elements include patient demographics, specific disease characteristics, level of disease activity, test results, treatments, and clinical outcomes. Registry data are used to support chronic care management reports that enable pre-visit planning, population management and patient-tracking, and comparative performance measurement (monthly charts displaying clinical, process, and outcome measures). Registry data can also be used to conduct various types of research, including comparative effectiveness studies. Centers provide their own resources to support data capture as part of their participation in the network.

### Development of the Quality Improvement Intervention to Enhance Data Quality

The ICN Data Management Committee was formed as part of the network’s data coordinating center at Cincinnati Children’s Hospital Medical Center to inform the design, development, and testing the data quality QI process. Representatives include staff from the data coordinating center for the network as well as individuals from participating ICN clinical care centers. The following disciplines are included on the committee: clinical medicine, research coordination, data management, biostatistics, clinical epidemiology, project management, and QI. The data coordinating center of the network reports to the network’s executive leadership and is an ongoing network function. The DCC and the committee are accountable for the ongoing maintenance and improvement of data quality. Its work is funded as part of network operations.

A structured QI process was used to design a data quality system with the following aim:

“*To assist ICN care centers in creating a reliable, accurate and consistent data collection system and full population registry (all patients, all visits, all data items) so patients can rely on the recommendations generated from the registry and physicians and researchers can use data to make decisions with confidence about the care and outcomes of children with IBD*.”

**Figure 1:**
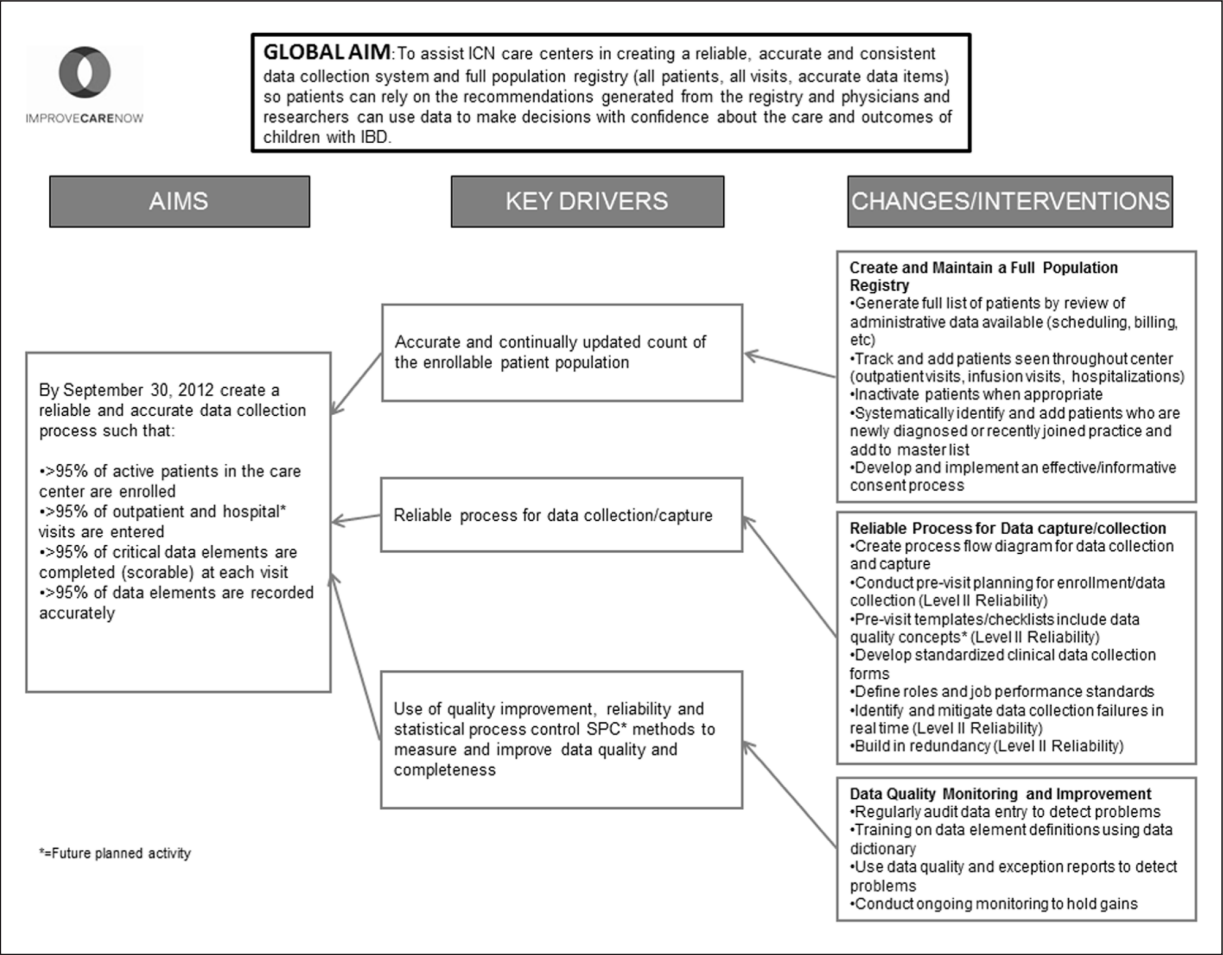
ImproveCareNow’s data quality key driver diagram.

A logic model known as a key driver diagram was developed to identify changes and interventions that support the drivers of good data quality (Figure 1). These key drivers included having consistent and timely information about the population, having reliable data collection practices, and the application of QI methodology to improve data quality. As illustrated in the key driver diagram, specific interventions in several processes were identified: creating and maintaining a full population registry, developing a reliable process for data collection, and monitoring data quality.

The data quality improvement effort was built upon ongoing QI training and support processes that were already established for centers. The team initially defined nine measures that measured completeness, consistency, and timeliness of data entered into the ICN registry (see Appendix A). These measures were implemented in April 2011 and the consistency measures were bundled together in late 2013 due to high performance on each measure and similarity of content. The measures still covered the same broad categories (completeness, consistency, and timeliness). Two additional measures for monitoring hospitalization data were added in 2014. Only current measures only are presented. The original data quality reports were developed to look like the reports for the clinical process and outcomes measures already utilized by centers and distributed on a monthly basis. By July 2013, the data quality charts were made available electronically in conjunction with the other QI tools in the system including clinical measures, population management reports, and pre-visit planning reports. In addition, the reporting system was modified so that the results were updated on a nightly basis to align with daily data collection updates. This enabled centers to view the charts at any time, whereas previously changes were not reflected until the next reporting month.

### Components of the Data Quality Intervention

#### Creating and Maintaining a Full Population Registry

A full population registry contains complete data for all visits for all patients in the registerable (target) population. Determining if all potential patients were included in the registry and if all visits had been entered could not be done solely by querying the registry but required additional work for the centers to identify and report their eligible population and evaluate which visits should be entered into the registry. To obtain an accurate and timely population total, centers must query clinical records to identify the number of eligible patients followed in the practice. Early efforts focused on developing center-specific procedures to identify their eligible population and subsequently providing this total to ICN on a quarterly basis. Centers with early success were asked to share the details of their process with all centers at network-wide meetings and webinars. This total population is now entered by each center into the registry on a quarterly basis. If they do not update the number, it carries forward. Using center reported denominators, the percentage of eligible patients with data in the registry is calculated. When a patient no longer participates in the registry, the center changes the status for that patient to “deactivated”. This can occur for numerous reasons including transferring to an adult gastroenterologist, moving, etc. To ensure that the patient population at each center is accurately represented, the percentage of patients with a visit in the past 13 months is calculated and monitored. This measure helps centers identify and remove patients no longer receiving care at the center. If a patient has not been seen, this could be an indication that they should be deactivated and should not be included as part of the practice population or the registry moving forward.

To estimate the percentage of visits captured in the registry each month, centers are asked to provide the visit dates and patient IDs for any registered patient having a visit during the first full week of each month. The percentage of visits captured in the registry for that week is then calculated and used to estimate the percentage of visits captured monthly.

Hospitalization is a serious outcome for any patient, and data quality is monitored using two mechanisms. The first is a measure assessing whether centers entered at least one hospitalization in the past 90 days. As the network has become more diverse, it has become apparent that the assumption that every center will have a hospitalization to enter every 90 days is not necessarily valid. To assess the timeliness of data entry for hospitalizations, a second measure was implemented, tracking the percentage of hospitalizations entered into the registry within 30 days of discharge. More recently, centers have been asked to start entering the total number of hospitalizations that occur each month to compare with the number entered, a process similar to how visits are tracked.

#### Monitoring Data Quality

In September 2011, network centers were asked to conduct a failure mode and effect analysis [13] of their data collection and capture process based on the data quality and exceptions reports. This exercise led centers toward the application of high reliability processes for data collection and capture including documenting the process of data capture with process flow diagrams. These tools were subsequently incorporated into training for centers joining the network. Ideas to improve data collection continue to be posted on a website that enables centers to share knowledge learned.

**Figure 2:**
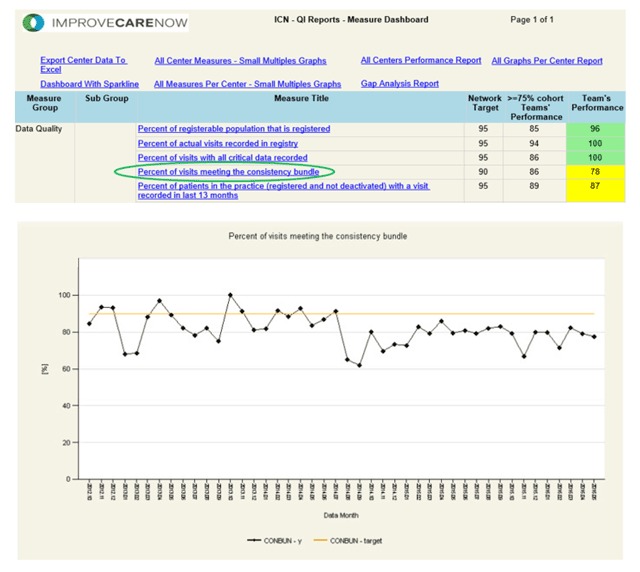
Sample center level data quality report as received by participating centers monthly.

Center-level and aggregate statistical process control charts were developed to show performance on each of the measures of data quality. A sample data quality report that ICN centers see in the registry is displayed in Figure 2. Measures are designed to assess completeness, timeliness, and consistency of the data. Four measures of completeness for population, outpatient visits, and hospitalizations were described above. An additional measure of completeness examines the data elements considered critical that are entered for each outpatient visit. These data elements are critical because they are required to determine disease activity and track medication use, key components of patient outcome data. Specifically, the set of critical variables include height, weight, medication data, Physician’s Global Assessment (PGA), and the individual components needed to score the short Pediatric Crohn’s Disease Activity Index (sPCDAI) [14] and Pediatric Ulcerative Colitis Activity Index (PUCAI) [15]. Two additional measures are used to assess timeliness: visits and hospitalizations entered within 30 days. Finally, consistency was evaluated using four measures. The first required age adjusted height, weight, and BMI z-scores to be between –4 and 4; the second required physician global assessment and PUCAI/sPCDAI to differ by no more than one classification category; the third required height to be no more than 1 centimeter less than it was on the prior visit; the fourth required changes in disease extent and phenotype to be clinically acceptable. Due to high performance on each of four measures, they were bundled, or combined into a single measure of intra- and inter-visit consistency, thereby requiring each of the four previous measures to be satisfied to pass the bundle measure. Data quality improvement training began in March 2011, and centers received their first data quality reports in May 2011. Center personnel were trained to monitor the data quality reports for trends to evaluate improvement efforts or to detect problems so that process failures could be identified and improvements implemented. New centers are trained in a similar manner during their onboarding process.

**Figure 3:**
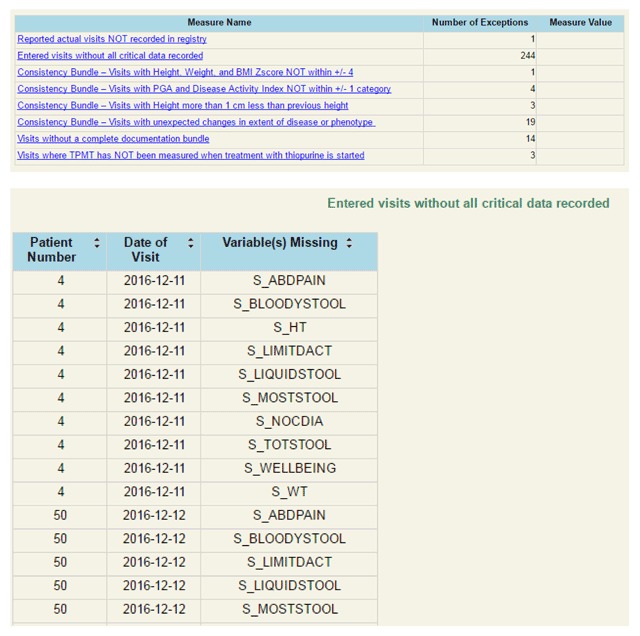
Sample exceptions report demonstrating center’s view of missing data elements.

To enable centers to easily identify data quality failures, a data quality “exceptions report” was developed, similar in appearance to the population management reports already in use by centers. These reports allow users easy access to specific information (a list of data elements that may represent an error) about each data quality failure. One example of a center’s exception report for the complete critical data measure is displayed in Figure 3.

Beginning in January 2015, the ICN Data Management Committee began monitoring data quality charts monthly for the network as a whole and on a quarterly basis for each center. Follow-up is conducted with centers to identify opportunities for shared learning or improvement. Centers are also reminded about data quality through routine calls with their quality improvement consultants and on network-wide webinars.

### Statistical Analysis

Statistical process control (SPC) methods were used to monitor changes in performance for each data quality measure. Control charts were used to analyze process improvement efforts and identify and classify causes of variation into common cause variation (inherent in the system) and special cause variation (variations not normally part of the system that arise due to specific events) [16]. Center lines were estimated and control limits were calculated as the center line proportion ±3 standard deviations. Each control chart was monitored for evidence of special cause changes using standard SPC rules [17,18,19]. Center-lines were shifted if a sustainable change had taken place based upon review by the ICN Data Management Committee taking into account SPC rules and subject matter expertise.

## Results

**Figure 4:**
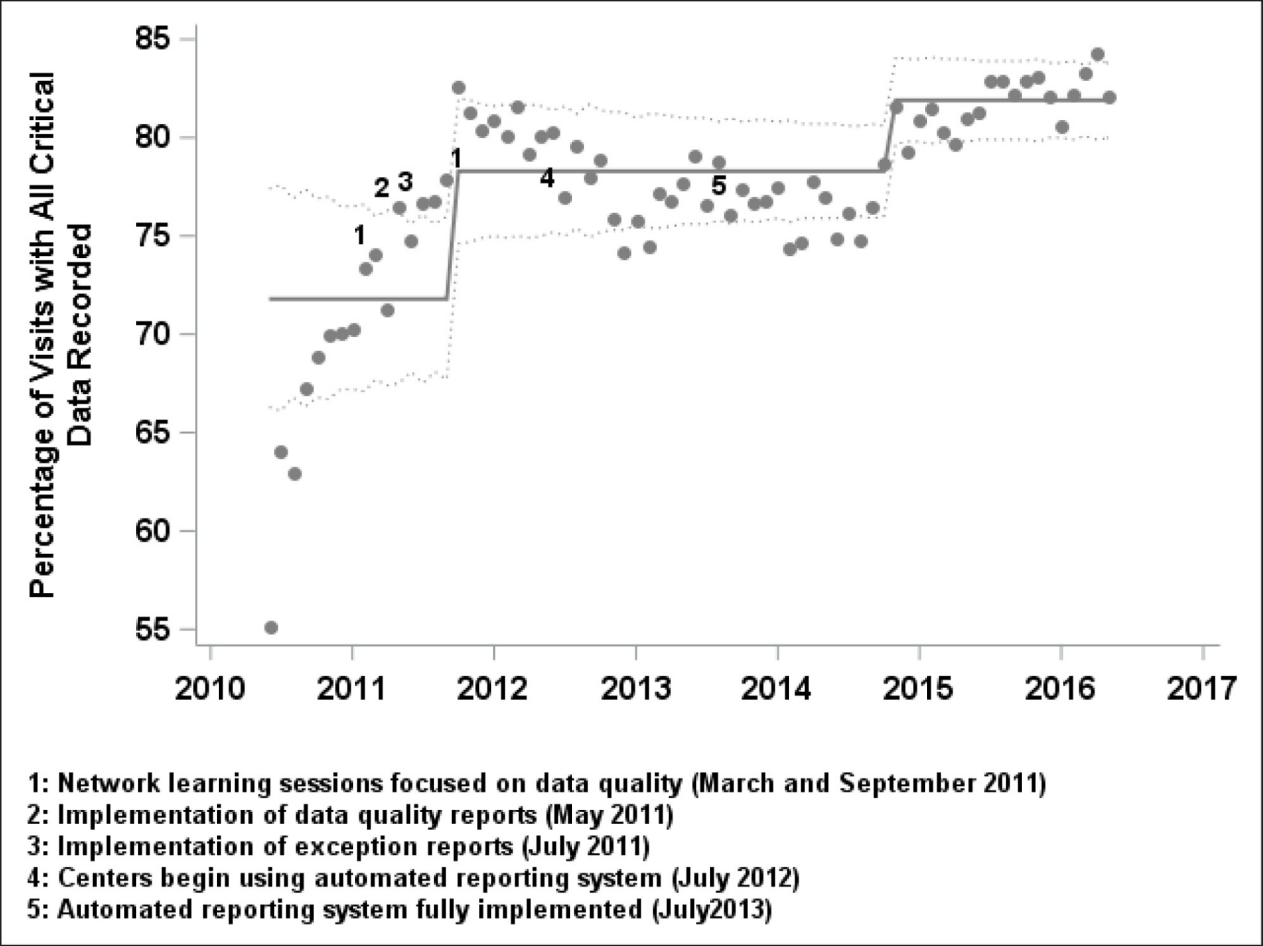
Annotated control chart displaying change in aggregate data element completeness.

**Figure 5:**
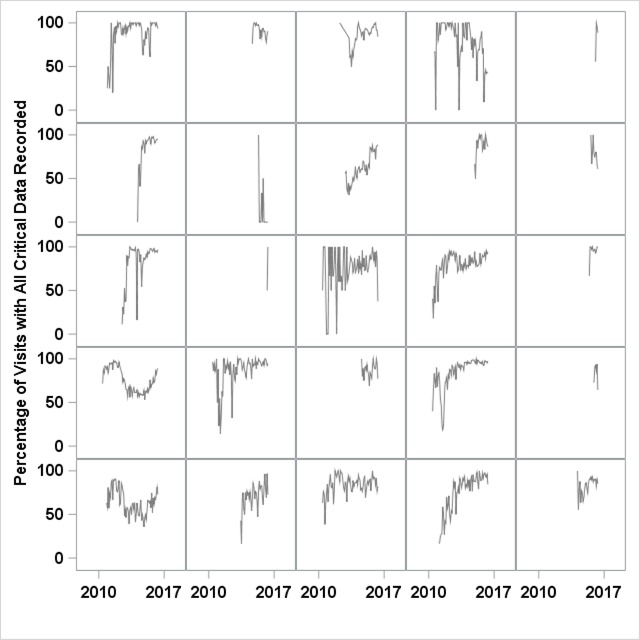
Random sample of 25 centers exposed to data quality reporting and training, showing intra-visit data completeness over time. For each center, the percentage of visits each month with complete critical data elements is indicated on the y-axis. The x-axis indicates June 2010 through June 2016.

By June 2016, 74 centers were participating in the ICN registry and had enrolled 24,309 patients and entered data from 162,626 visits. There was a pattern of improvement across 8 measures of data quality. The percentage of registered patients increased from 59 percent to 83 percent, with 51 percent of centers having registered at least 90 percent of their IBD population. Overall, 92 percent of visits of registered patients were recorded. In October 2011, there was evidence of a significant improvement in the percent of visits with all critical variables recorded based on the SPC criterion of one point outside of the control limits (Figure 4). By November 2014, there was evidence of additional improvement. Based on this special cause and subject matter expertise indicating purposeful changes to the system of data quality, the center line and associated control limits were shifted from 72 percent for the period June 2010 to September 2011 to 78 percent for the period October 2011 to October 2014, and again to 82 percent for the period November 2014 to June 2016. Figure 5 is a small multiple display of the complete critical data measure for participating individual centers. This display highlights the variability among centers for this measure. Some of the centers had high data quality across all time points.

Improvements in data quality over time can be seen in other measures. From June 2010 to June 2016, the completion rate for PUCAI increased from 73 percent to 91 percent and that for sPCDAI increased from 60 percent to 68 percent. The bundled measure of intra- and inter-visit consistency of the data (Appendix A) demonstrated common cause variation. During this time there was a change in the definition of extent of disease from the Montreal classification to the Paris classification.

The percentage of visits entered within 30 days of visit date varied widely over time but demonstrated improvement as well. New chronic care management reports (i.e. population management and pre-visit planning) were implemented in 2012 that required timely data entry for their effectiveness, leading to improvements in this measure. The measure of patients active in the registry with a visit in the past 13 months increased over time from 89 percent to 91 percent.

By June 2016, the percentage of centers having at least one hospital discharge in the past 90 days was 82 percent and the percentage of hospitalizations entered into the registry within 30 days of discharge improved from 42 percent to 63 percent.

## Discussion

We found that efforts to improve data quality that included training in quality improvement methods, and tools including control charts, exception reports and failure mode and effect analysis resulted in improvement in a range of data quality measures. These measures have continued to improve since 2016. This improvement was augmented by the development of electronically available control charts and reports that were updated daily and available to the centers on demand. We also observed variation among centers in data quality that could be due to the variation in use of these tools by a center. Measures of completeness of critical data and the percentage of patients registered increased. Measures of consistency and the percentage of visits recorded in the registry remained at their high baseline levels.

The success of the ICN registry rests on the QI training participating centers had already received to standardize clinical care processes to improve outcomes. Compared to comprehensive data cleaning that is typically used for clinical trials, this approach is much less labor intensive and requires fewer resources. Our approach is conceivably more sustainable given the focus on changing processes to improve data quality at the point of data entry instead of correcting errors after entry takes place.

Several authors have discussed the importance of high data quality in data registries [20,21,22,23,24,25,26]. Previous studies have emphasized methods to assess the quality of data in registries, but there has been little work on models to improve registry data quality that go beyond centralized data quality support. Kahn et al [24] proposed several data quality assessment methods to gauge single-site data quality that include rules for evaluating single-item completeness and validity (e.g. toddler’s height cannot be 7 ft.) and cross-item temporal (e.g. surgery date cannot be after death date) and relational consistency (e.g. males cannot have positive pregnancy test). The authors also discussed analyzing temporal data to evaluate logical consistency relative to the evolution of a process or set of states over time and the examination of conditional dependencies based on knowledge of a clinical scenario (e.g. gestational period for human cannot be 48 months). Brown et al [22] discusses best practices and provides recommendations for data quality checking in distributed data networks. In a distributed data network, a common data model is developed to standardize the content and format of observational data that facilitates cross-site analyses. Data are checked to ensure they conform to the common data model (e.g. valid values for SEX “M”, “F”, “U”). In addition to evaluating conformity with the data model, Brown states that additional checks should be done by evaluating individual item completeness and validity and cross-item temporal and logical consistency [22]. Despite differences in methodology, both aforementioned groups recommend making information about data quality of a database available to users of the database. To our knowledge, this is the first report detailing an effort to improve data quality using quality improvement methods and additional tools described in this manuscript.

A limitation of this study is that centers were not randomly assigned to the data quality QI support intervention. Although it is possible that improvement could have taken place on its own, prior to the intervention, the measures were stable over a long period of time, and we did not observe a special cause signal until the implementation of the data quality program. This observation is limited by the inability to track the use of the data quality tools. However, this is no longer a problem as the reporting system has gone online and now has usage tracking capabilities.

The ICN Data Management Committee provides a structure to enable the network’s focus on improving data quality to evolve over time. Ongoing monitoring of data quality is part of the network’s monthly dashboard of measures. The automated assessment of data quality measures allows the network to monitor data quality over time and to detect changes in data quality that need further exploration as well as to inform the ongoing improvement of the measures themselves.

Complete and accurate registry data are essential for managing chronically ill patients, guiding improvement efforts, and research. As the emphasis on multi-site registries and networks increases, so will the importance of their data quality. Future work should focus on designing, developing, and testing additional interventions to further increase the quality of registry data.

By raising awareness of the importance of high data quality and supporting member centers’ use of QI training and tools, the completeness and consistency of data within the ICN registry has increased. In conclusion, our work demonstrates that QI approaches to improving data quality are effective and feasible to implement in research and improvement networks. Furthermore, this results in a higher level of confidence when accessing the data for various purposes, including clinical decision making.
